# Moyamoya Disease Diagnosed With Intracranial Hemorrhage After Cesarean Section Under Spinal Anesthesia: A Case Report

**DOI:** 10.7759/cureus.56436

**Published:** 2024-03-19

**Authors:** Yasir Ilyas, Kıvanç Öncü, Kübra İlyas, Ahmet Beşi̇r

**Affiliations:** 1 Anesthesiology and Reanimation, Trabzon Fatih State Hospital, Trabzon, TUR; 2 Anaesthesia and Reanimation, Sinop Atatürk State Hospital, Sinop, TUR; 3 Anesthesiology and Reanimation, Karadeniz Technical University, Trabzon, TUR

**Keywords:** caesarean section, spinal anesthesia, intraventricular hemorrhage, postpartum, moyamoya disease

## Abstract

Moyamoya disease (MMD) is a rare non-inflammatory cerebral vasculopathy characterized by progressive stenosis of the internal carotid arteries, often bilaterally, and the formation of abnormal collateral vascular structures at the cranial base. A patient who underwent elective cesarean section (C/S) twice under spinal anesthesia and was diagnosed with MMD as a result of recurrent intracranial hemorrhage in the postpartum periods is presented.

A 41-year-old female patient without any systemic comorbidity, gravida 2, parity 2, had her second cesarean section (C/S) operation under spinal anesthesia and was discharged on the third postoperative day without any problems. The patient had a mild headache that started from the occipital region and spread to the entire cranium on the same day. After applying to the emergency department at different times, she was discharged with conservative treatment. The patient had a severe headache and was admitted to the emergency room on the ninth postoperative day. The patient, who was diagnosed with intracranial hemorrhage after cranial imaging, was referred. Cranial angiography revealed advanced bilateral internal carotid artery symmetric occlusion and the basilar artery was preserved. According to the angiographic image, the patient was diagnosed with moyamoya disease and was followed up in the intensive care unit. The muscle strength of the patient, who had no cranial nerve pathology or lateralization findings, was evaluated as normal. Conservative management was applied in the intensive care unit. The patient was discharged with recommendations for neurosurgery and cardiovascular surgery after 12 days.

In the postpartum period, especially in cases of headache that persists for a long time after dural puncture and does not have a postdural feature, intracranial hemorrhage should be considered until proven otherwise, and moyamoya disease also be considered in the differential diagnosis of intracranial hemorrhage. The approach to the patient in the perioperative period should focus on providing normotension, normocapnia, normothermia, and effective analgesia.

## Introduction

Moyamoya disease (MMD) is a rare non-inflammatory cerebral vasculopathy characterized by progressive stenosis of the internal carotid artery, often bilaterally, and the formation of abnormal collateral vascular structures at the cranium base [1]. The etiology of MMD is not yet clear. Incidence is higher in women, especially in the second and third decades. Women are especially likely to be diagnosed during their reproductive years, and this situation complicates the pregnancy period [2]. In a review published in 2018, it was reported that the diagnosis of MMD in pregnant women was detected in three different periods: before pregnancy, during pregnancy, and after pregnancy [3]. Although the clinical features of MMD vary with age, in children, it mainly includes symptoms such as epileptic seizures, motor paralysis, speech impairment, and retardation of intellectual capacity due to transient ischemic attack or cerebral infarction. However, in adults, the main symptoms are headache and/or impaired consciousness due to cerebral hemorrhage or cerebral ischemia [4]. We present the case of a patient who was diagnosed with MMD as a result of recurrent intracranial hemorrhage in the postpartum periods after two elective caesarean sections under spinal anesthesia.

## Case presentation

**Figure 1:**
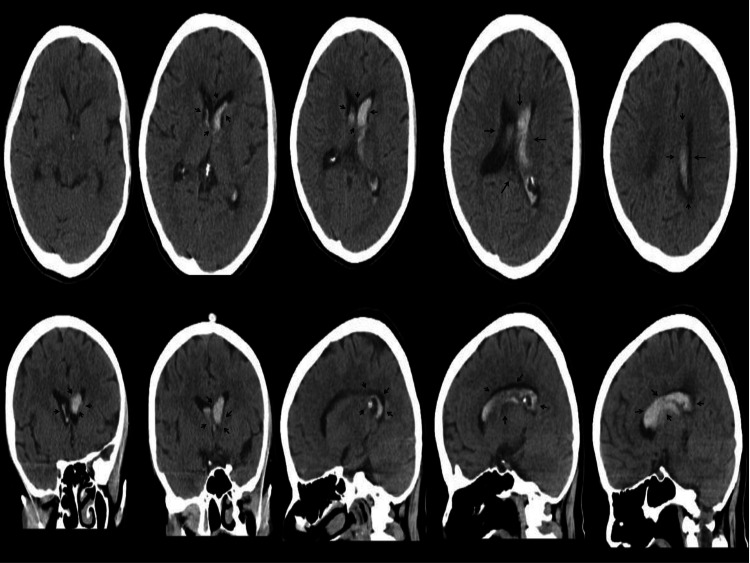
Axial, Coronal and Sagittal Plan View of Intraventricular Hemorrhage Hemorrhagic areas are marked on the figure with black arrows. Top - axial section; bottom - two images from left: coronal section; three images from right: sagittal section

A 41-year-old female with no systemic comorbidity had her second cesarean section (C/S) under spinal anesthesia and was discharged on the third postoperative day. On the same day, she had a mild headache, starting from the occipital region and spreading to the entire cranium. The patient, who was examined by a family physician on the seventh postoperative day, was referred to a health center for further cardiac examination upon being evaluated as hypertensive, but no pathology was detected. She was referred to the emergency department with a severe headache on the ninth postoperative day, and subarachnoid hemorrhage (SAH) was detected in the cranial radiological imaging (Figure 1). There were no neurological findings in the physical examination of the patient, except for severe headache.

**Figure 2:**
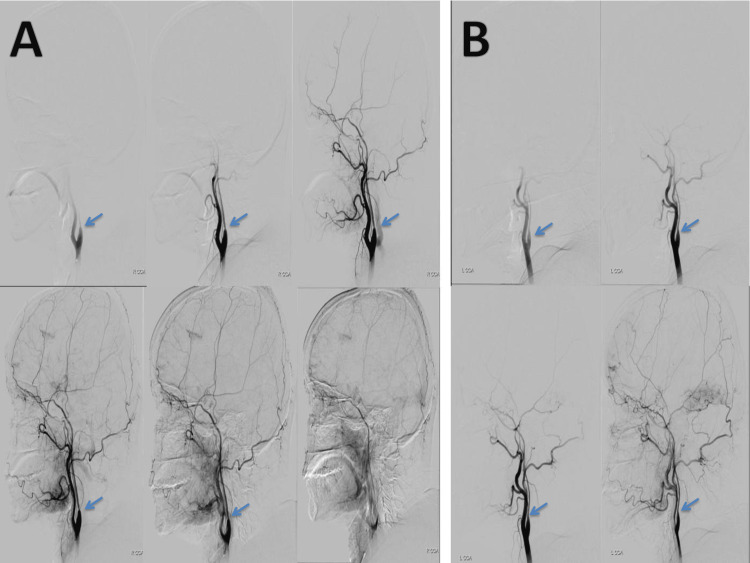
Digital Subtraction Angiography Image The bilateral internal carotid artery is severely narrowed symmetrically from the cervical segment to the supraclinoid segment, and there is total stenosis starting from the ophthalmic artery level. Both anterior and middle cerebral arteries and their branches appear non-opaque. Weak opacification is observed with leptomeningeal collaterals from bilateral external carotid artery branches. (Regions with major narrowing are marked with a blue arrow. A: right carotid artery B: left carotid artery.)

Digital Subtraction Angiography (DSA) was performed on the patient to evaluate the vascular structures and investigate the etiology. According to the DSA imaging, advanced bilateral internal carotid artery symmetric occlusion was detected and the basilar artery was observed to be preserved (Figure 2). As a result of the angiographic findings, the patient was diagnosed with stage 5-6 moyamoya disease according to the Suzuki Classification and was followed up in the intensive care unit.

When her medical history was questioned in detail, it was learned that she had a history of SAH (symptoms in the postpartum period; severe headache, speech impairment and drowsiness) after a C/S performed under spinal anesthesia in 2012. The etiology was not investigated and she was discharged without neurological sequelae after 15-20 days of conservative treatment. The patient had no history of medicine or addiction.

It was learned that spinal anesthesia was applied to the patient after a dural puncture in the first attempt at the lumbar 3-4 level in a sitting position. When the lumbar spine was examined, no cerebrospinal fluid (CSF) leak was detected at the macroscopic level. The patient was evaluated as conscious, cooperative and oriented (Glasgow Coma Scale (GCS):15). The muscle strength of the patient, who had no cranial nerve pathology or lateralization findings, was evaluated as normal.

A Conservative management approach was applied during intensive care unit follow-up. On the third day in ICU, the patient, whose cranial hemorrhage had regressed and whose clinical condition remained stable, was transferred to the neurosurgery ward. After 9 days of follow-up, medical treatment was arranged (phenytoin tab 100 mg 3x1, paracetamol tab 500 mg tb 3x1), and neurosurgery and cardiovascular surgery routine check-up was recommended. Afterwards, the patient was discharged uneventfully.

## Discussion

Headache is a common problem in the postpartum period; the incidence has been reported as 39% [5]. Differential diagnoses of headache after dural puncture include postdural puncture headache (PDPH), migraine, pneumocephalus, and intracranial pathologies [6].

PDPH occurs as early as 12 hours after lumbar puncture and generally within 5 postoperative days [5]. There may be diagnostic delays of intracranial hemorrhage cases because the symptoms are similar to processes such as PDPH.

The incidence of hemorrhage associated with neuraxial anesthesia is quite low and has been reported to be more common after epidural anesthesia (1:150000) compared to spinal anesthesia (1:220000) [7]. Reported cases of intracranial hemorrhage after lumbar puncture in obstetric patients is limited. Considering the literature, there are articles reporting intraspinal and/or intracranial subarachnoid, subdural and intraparenchymal hemorrhage after spinal anesthesia [8-11].

It has been stated that intracranial hemorrhage develops as secondary to moyamoya, due to microaneurysms and/or pathological neoangiogenesis-related arteriovenous malformations secondary to carotid artery stenosis [12]. In addition to neoangiogenesis, physiological changes that occur during pregnancy, such as increased blood volume in the circulatory system and hypercoagulation, may also increase the risk of neurological comorbidities in moyamoya disease [4].

Due to current pathophysiological processes, it has been reported that women are at high risk for ischemic/hemorrhagic cerebrovascular comorbidities, especially during pregnancy, and therefore the maternal mortality rate may be 35-83% [13]. Mortality and morbidity rates may increase further as a result of intracerebral vascular malformations.

It has been observed that cerebrovascular comorbidities increase in patients with MMD within the first 6 weeks of the postpartum period [14]. In a review, it was concluded that 66.6% of patients diagnosed with MMD in the postpartum period were detected within the first week and were frequently diagnosed secondary to ischemic cerebrovascular events [3].

There is insufficient literature on uneventful birth in MMD patients. Besides that, there may be many undiagnosed MMD patients who give birth without developing peripartum complications. Pathological vascular structures defined as moyamoya vessels, which can cause impaired autoregulation of cerebral blood flow, may rupture as a result of stimuli affecting cerebrovascular hemodynamics.

There is no certain consensus on the recommended anesthesia technique in cases with MMD. However, there are publications reporting that general anesthesia or neuraxial anesthesia techniques can be applied successfully. Anesthesia management depends on maintaining the balance between cerebral metabolic oxygen consumption rate (CMRO2) and cerebral blood flow (CBF) to prevent neurological morbidity. As a conclusion, the fundamental approach in the perioperative period should be to ensure normotensive, normocapnic and normothermic conditions.

It has been observed that CBF in MMD patients is lower than in healthy individuals, and decreases in mean arterial pressure (MAP) cause cerebral hypoperfusion in direct proportion to CBP. Therefore, keeping MAP at an optimal level is important for effective CBF [15].

Carbon dioxide has important effects on cerebral vascular tone. It is obvious that significant decreases in CBF may occur due to hypocarbia. It has also been reported that hypercarbic vasoreactivity is reduced in adults with MMD and that hypercarbia may have negative effects on CBF [16]. It should be kept in mind that, in addition to the other effects, hypothermia may also cause deterioration in CBF by accelerating vasospasm.

Although the general anesthesia approach has advantages such as reducing cerebral metabolic oxygen consumption, it also has disadvantages in terms of aspiration risk, neonatal depression and impaired cerebral autoregulation. In addition, hemorrhage may occur as a result of increased cerebral metabolic rate and rupture of intracranial vascular pathologies after the stress response caused by general anesthesia processes such as intubation, insufficient depth of anesthesia, and extubation [17].

It has been reported that labor has been successfully performed in many cases with neuraxial anesthesia techniques. In addition, neuraxial techniques have advantages such as continuous monitoring of the state of consciousness and early detection of complications [17].

Postoperative pain management is another important consideration. Postoperative pain is acute pain that begins with surgical trauma and gradually decreases as the tissue heals. It may last up to 3 months in the postoperative period [18]. While postoperative pain can be experienced in different presentations depending on the person, a decrease in CBF may occur as a result of the anxiety-hyperventilation-hypocapnia cycle secondary to postoperative pain. As a result, the risk of cerebrovascular comorbidities may increase. Therefore, it is important to maintain the effective analgesia provided with care during hospitalization, even after discharge.

It is known that ischemic cerebrovascular comorbidities are often observed in the postpartum period of MMD patients. The present case differs in terms of MMD presentation due to the presence of intracranial hemorrhage in the postpartum periods of both pregnancies. Intracranial hemorrhage may be caused by various reasons. Although the main possible cause is pathological angiogenesis due to MMD, intracranial hypotension secondary to CSF leak after dural puncture, ineffective postoperative analgesia, and pregnancy-related hematological changes may also have contributed to the rupture of fragile vascular structures.

## Conclusions

For all the reasons discussed, the patients and their relatives should be informed in the preoperative period, considering that hemorrhage may recur in the postpartum period in MMD patients with a history of intracranial hemorrhage. In a nutshell, in the postpartum period, especially in cases of headache that persists for a long time after dural puncture and does not have a postdural feature, intracranial hemorrhage should be considered until proven otherwise, and moyamoya disease also be considered in the differential diagnosis of intracranial hemorrhage.
